# Generation of Mechanical Characteristics in Workpiece Subsurface Layers through Milling

**DOI:** 10.3390/ma17071552

**Published:** 2024-03-28

**Authors:** Michael Storchak, Larysa Hlembotska, Oleksandr Melnyk

**Affiliations:** 1 Institute for Machine Tools, University of Stuttgart, Holzgartenstraße 17, 70174 Stuttgart, Germany; 2 Department of Mechanical Engineering, Zhytomyr Polytechnic State University, Chudnivska Str. 103, 10005 Zhytomyr, Ukraine; zid_gle@ztu.edu.ua (L.H.); o.l.melnyk@ztu.edu.ua (O.M.)

**Keywords:** cutting, milling, subsurface layers, nanoindentation, sclerometry, indenter penetration work, indenter penetration depth

## Abstract

The generation of mechanical characteristics in workpiece subsurface layers as a result of the cutting process has a predominant influence on the performance properties of machined parts. The effect of the end milling process on the mechanical characteristics of the machined subsurface layers was evaluated using nondestructive methods: instrumented nanoindentation and sclerometry (scratching). In this paper, the influence of one of the common processes of materials processing by cutting—the process of end tool milling—on the generation of mechanical characteristics of workpiece machined subsurface layers is studied. The effect of the end milling process on the character of mechanical property formation was evaluated through the coincidence of the cutting process energy characteristics with the mechanical characteristics of the machined subsurface layers. The total cutting power and cutting work in the tertiary cutting zone area were used as energy characteristics of the end milling process. The modes of the end milling process are considered as the main parameters affecting these energy characteristics. The mechanical characteristics of the workpiece machined subsurface layers were the microhardness of the subsurface layers and the total work of indenter penetration, determined by instrumental nanoindentation, and the maximum depth of indenter penetration, determined by sclerometry. Titanium alloy Ti10V2Fe3Al (Ti-1023) was used as the machining material. Based on the evaluation of the coincidence of the cutting process energy characteristics with the specified mechanical characteristics of the machined subsurface layers, the milling mode effect of the studied titanium alloy, in particular the cutter feed and cutting speed, on the generated mechanical characteristics was established.

## 1. Introduction

The most common methods of ensuring the required service properties of parts for various machines and mechanisms are various chip formation–cutting processes. As a result of the tool’s thermomechanical impact on the machined workpiece, certain mechanical properties are generated in the workpiece’s subsurface layers [1,2]. These properties, evaluated in terms of the hardness parameters (including microhardness) [3], residual stresses [4,5], material structure [6,7] and others, have a decisive influence on the service properties of machined parts and their durability [8]. The formation patterns of these mechanical properties are essentially determined by the machining process conditions, which in turn are regulated by the used cutting modes. Numerous publications have been devoted to the study of the relationship between the formation regularities of mechanical properties of subsurface layers and the machining process conditions. In the past few decades, this research focus has also been supported by numerical modeling [9]. This significantly expands the possibilities and application area of this research field, as well as providing a significant reduction in the cost of experimental research. In particular, it is relevant in the study of mechanical property generation patterns in subsurface layers of complex-profile parts [10,11] with the use of spatial cutting processes. Spatial cutting processes are characterized by a significant variability in the contact conditions between the tool and the machined material, of which end milling is a striking representative, generating a significant gradient of mechanical properties in the subsurface layers, which is all the more aggravated in the case of hard-to-machine metals and alloys [12]. These circumstances cause significant difficulties both in evaluating the above mechanical properties and in establishing their relationship with the cutting process conditions. Significant support in the evaluation of mechanical characteristics is provided by micro- and nanometer methods, in particular, instrumental nanoindentation [13,14] and the sclerometry (scratching) of surfaces [15,16], ensuring the determination of integral mechanical characteristics, which are quite closely related to the conditions of their generation, in this case, the cutting processes [17].

The present study is devoted to the investigation of the relationship between the mechanical characteristics of subsurface layers generated during the end milling of ß-phase titanium alloy Ti10V2Fe3Al (Ti-1023) and the cutting process conditions.

## 2. Brief Description of the State of the Art on the Determination of Mechanical Characteristics from Machined Subsurface Layers

The most commonly measured mechanical characteristics of machined subsurface parts include microhardness, residual stress magnitude, and the microstructure of the machined material. These characteristics are also used to evaluate the mechanical properties of the workpiece subsurface layers of hard-to-machine metals and alloys subjected to milling [18]. Dai et al. [19] studied the hardening process of machined subsurface layers from the Inconel 718 workpiece. They established the effect of the cooling method in the cutting zone on work hardening. Analyzing the effect of cutting speed and tool feed on the work hardening of the same machined material was the focus of a study by Ren and Liu [20]. As a result, the optimal cutting modes were determined, providing the required hardening of machined subsurface layers. Investigating the cutting process of Inconel 718 at different tool rake angles and the machined material structure, Xu and colleagues [21] determined the relationship between the geometric parameters of the tool and the mechanical characteristics of the workpiece’s machined subsurface layers. The deformation value of the subsurface layers and their hardening value were used as the studied mechanical characteristics. The study of the microhardness formation process in the subsurface layers during the micro milling of nickel alloy Inconel 718 depending on the cutting modes, in particular, cutting speed, cutter feed, and axial cutting depth, was undertaken by Lu et al. [22]. Xavior and colleagues [23] examined the formation mechanism of Inconel 718 hardening, as well as the formation of residual stresses in it and its microstructure under different tool materials and cutting conditions. The influence of alternative machining processes on surface integrity and the regularities of residual stress formation in the machined subsurface layers of alloy 718 are the subject of a study by Suárez et al. [24]. The formation of machined surface microhardness of titanium alloy Ti-6Al-4V is the subject of studies by Hou with Li [25] as well as Mathoho and coworkers [26]. Monka and colleagues [27] considered the influence of cutting modes and tool geometric parameters on the microhardness of the machined surface in orthogonal and oblique cutting. They obtained response surfaces and correlation dependences of microhardness on the above-studied parameters. The microhardness prediction of the milling machined subsurface layers is the subject of a study by Wang [28]. He used different methods of regression analysis in carrying out this process. The residual stress formation in the subsurface layers of such difficult-to-machine materials as duplex steel and titanium alloy Ti-6Al-4V as a result of the cutting process was studied by dos Santos and colleagues [29] and Rangasamy et al. [30]. A considerable amount of research on the mechanical characteristics of surface layers machined by cutting is devoted to the analysis of the machined material microstructure and the cutting process conditions’ influence on it, as found in [31]. At the same time, the microstructure of the machined material was evaluated based on microhardness distribution. Thus Mendas et al. [32] and Ameri with colleagues [33] determined dislocation density by measuring microhardness distribution. Among other mechanical characteristics of the subsurface layers, Alijani et al. [34] studied the microstructure of titanium-nickel alloy after milling. As a result, they determined the effect of machining process conditions on the formation of the studied microstructure. The study by Chen and coworkers investigates the effect of cutting depth and the corresponding magnitude of plastic deformation of Inconel 690 nickel alloy subsurface layers as a result of milling [35]. Haddag and colleagues [36] studied the influence of cutting modes during the machining of titanium alloy Ti-6Al-4V, mainly the cutting speed and tool feed, on the formation of the machined subsurface layer structure. The need to determine the optimal cutting modes for the milling of nickel alloy Inconel 625, providing the necessary microstructure of the machined material, is reported in the study of da Silva et al. [37]. At the same time, the form parameters of the relative movement of the tool and the workpiece, which have the greatest influence on the microstructure of the machined material, were determined. Rajguru and Vasudevan [38] studied the effect of the Inconel 625 milling process on the microhardness of subsurface layers when machining without coolant with coated milling cutters. The influence of both the strain hardening and thermal softening of the machined surfaces was considered. It should be noted that in addition to evaluating the mechanical characteristics of the workpiece’s subsurface layers, a significant part of the studies on the surface integrity of difficult-to-machine materials is devoted to investigating the microtopography and, consequently, the microgeometry of the machined surface and the influence of cutting modes and conditions on microtopography parameters (see, for example, [39]).

Further development of the methodological and instrumental base for evaluation methods of the physical and mechanical characteristics of surfaces contributed to the creation of nondestructive testing methods for various surfaces and, in particular, for machined subsurface layers of parts. In this way, micro- and nanometric methods were established, in particular, instrumented nanoindentation [40,41,42], and sclerometry (scratch test) [43,44]. The research and development carried out by Atkins and Tabor [45] served as a prototype for the creation of an instrumented nanoindentation method. Further improvement of the method, using the continuous penetration of the indenter into the test material through the development of new devices and algorithms for evaluating the measurement results [46,47], ensured the creation of the currently widely used method of instrumented nanoindentation (see, e.g., [48,49]). Almost simultaneously with the instrumented nanoindentation method, the sclerometry method was established [15]. This method ensures the qualitative evaluation of coatings [16,50], such as coatings of carbide inserts used as cutting elements of various tool types, by determining the contact adhesion of the coating with the substrate [51,52]. A part of the uncertainty in the estimation of the indenter load at the moment of the studied surface fracture [53] has been recently compensated for through the use of the multi-pass scratching method [54,55]. The improvement and further development of the instrumented nanoindentation method and sclerometry depend predominantly on the instruments and devices that enable the realization of these methods. Therefore, a significant part of the studies devoted to these nondestructive testing methods is related to the creation and improvement of devices. In this regard, it is necessary to mention the study of Li et al. [56], devoted to the improvement of the calibration method of the device for instrumented nanoindentation through the use of an optical interferometer; the study of Peng et al. [57], devoted to the creation of a device for the realization of nanoindentation on subsurface layers, providing a significant increase in the accuracy of measurements; the work of Ding and colleagues [58], devoted to the study of different calibration methods of measured values; and the study of Fritz and Kiener [59], evaluating the influence of the environment of the device on the measurement results. An important role in the process of the further development of instrumented nanoindentation and sclerometry methods is played by research aimed at improving the methodology for evaluating the determined mechanical properties of materials. Such studies include works devoted to improving the stability of measurement results [60], the selection of an optimization method for determining mechanical properties [61], the analysis of contact stiffness fluctuations in the studied materials [62], the influence of the indenter contact conditions with the tested material on the measurement results, and many others. In this connection, it is necessary to point out the study of Harsono et al. [63], devoted to the investigation of the friction effect in the specified contact; the study of Wang, on establishing the relationship between the indenter penetration modes and the friction value in the contact with the measured mechanical properties [64]; the work of Sivaram et al., devoted to the study of the relationship between pile-up or sink-in effects and the strain hardening of the tested material [65]; and the investigation of friction phenomena in contact with synthesized materials through instrumental nanoindentation and sclerometry by Farayibi and colleagues [66].

The development of the instrumented nanoindentation method was mainly aimed at determining the microhardness of subsurface layers (see, e.g., [67,68,69]), the deformation degree of variously formed parts (see, e.g., [50,58,70]), the microstructure of materials (see, e.g., [71,72,73]), and the residual stresses in the subsurface layers of specimens generated by their previous formation (see, e.g., [74,75,76]). The determination of the material mechanical properties using the sclerometry method was also used somewhat later to investigate the microstructure and hardening of materials by estimating the indenter penetration depth into the test material (see, e.g., [77,78,79]). Recently, the tool indentation method and the sclerometry method have been used not only to determine the above-mentioned individual mechanical characteristics of the parts’ subsurface layers subjected to various machining processes but also to evaluate the integral (energy) mechanical characteristics [17,80]. In this regard, it is necessary to note the study of Bezyazychnyy et al. [81], devoted to the establishment of the relationship between cutting modes and physical and mechanical characteristics of the machined material, and the specific accumulated strain energy of this material along the workpiece depth. The relationship between the elastic and plastic components, as well as the total indentation energy and microhardness of the investigated specimens was the topic of the study of Yamamoto et al. [82]. The relationship between the mechanical properties of carbide metals and alloys and the cutting process conditions has been studied by Wang and coworkers [83] and Ren and Liu [20].

The analysis of the possibilities of the instrumented nanoindentation method and the sclerometry method shows the perspectives of these methods’ application to the determination of the mechanical characteristics of specimens’ subsurface layers, especially specimens from hard-to-machine materials, subjected to machining by cutting with essentially changing conditions of contact between the tool and the workpiece.

Simultaneously with the formation of a specified macro- and microgeometry of the machined part, the cutting process generates certain physical and mechanical characteristics in the machined subsurface layers of the workpiece. Under all other equal conditions, the value and distribution of these characteristics in the machined workpiece volume are mainly determined by the thermomechanical conditions of contact between the tool and the workpiece. In this regard, there are significant difficulties in determining these contact conditions in real three-dimensional cutting processes and, in particular, when machining difficult-to-machine materials. At the same time, such conditions are attracting the most interest from the industry. Taking into account this interest, a study of the influence of the milling process with end milling cutters on the formation of physical and mechanical characteristics of the workpiece subsurface layers was carried out, and titanium alloy was used as the machined material. This study is a continuation and methodological development of a previously published paper [17]. In the previous study [17], a coincidence of cutting power and cutting work with the mechanical characteristics of subsurface layers formed during the orthogonal cutting of structural steel was established. The cutting power and cutting work in the orthogonal cutting process were determined for different cutting speeds and tool rake angles. The mechanical characteristics of the machined subsurface layers were characterized by the total work of indenter penetration during tool nanoindentation and the maximum depth of indenter penetration during sclerometry.

## 3. Materials and Methods

**Figure 1:**
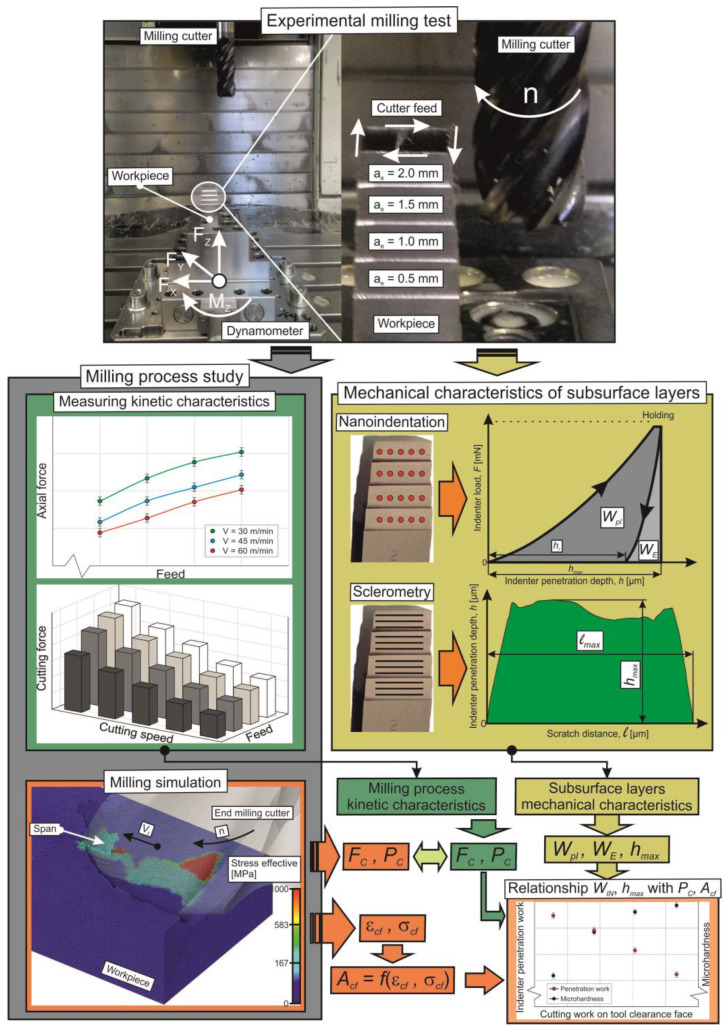
Methodology scheme for the determination of physical and mechanical characteristics of subsurface layers machined by milling.

The methodology for performing the present study to determine the physical and mechanical characteristics of the subsurface layers generated by the end milling process is explained by the scheme presented in Figure 1.

The initial stage of determining the physical and mechanical characteristics of the workpiece subsurface layers machined by the end cutter is to carry out experimental studies of the cutting process characteristics. These characteristics include the set of characteristics by which the cutting process expresses itself externally. Typically, these include kinetic characteristics, such as the cutting force components and their power, thermal characteristics, such as cutting temperature, heat flows in the workpiece and tool, stresses and strains in the tool-machined material contact pair, etc. In the present study, the kinetic characteristics of cutting were determined, namely, the resultant cutting force *F_C_* and the total cutting power *P_C_*. The next step is devoted to measuring the mechanical characteristics of the milled subsurface layers of the workpiece. These characteristics include the microhardness of the machined layers, the indenter penetration total work, and the maximum depth of indenter penetration [17,55]. The first two mechanical characteristics are measured using instrumented nanoindentation, and the last characteristic is determined by the sclerometry of the workpiece machined surfaces. In parallel with the first two steps, a simulation of the end milling process is performed through a numerical cutting model. The adequacy of the milling numerical model is verified by comparing the measured and simulated values of cutting force and cutting power. After that, a simulation of the studied machining process is performed, as a result of which the stresses *σ_cf_* and strains *ε_cf_* of the machined material in the area of the tertiary cutting zone are determined. These stresses and strains are subsequently used to calculate the thermomechanical effect of the tool on the machined material *A_cf_* in the specified cutting zone. This cutting work in the tertiary cutting zone *A_cf_* mainly determines the physical and mechanical characteristics of the machined subsurface layers of the workpiece. The subsequent and last stage of the research methodology for determining the physical and mechanical characteristics of the subsurface layers generated by the end milling process is devoted to analyzing the coincidence of the mechanical characteristics measured at the previous stage with the energy characteristics of the milling process: the total cutting power *P_C_* and the work of thermomechanical impact of the tool on the machined material *A_cf_* in the tertiary cutting zone. The presented methodology of the integral mechanical characteristics evaluation in the workpiece subsurface layers formed as a result of the milling process and the study of the coincidence between these characteristics and the energy characteristics of the cutting process influencing them will provide a deeper understanding of the physical processes that generate the mechanical properties of the machined surfaces of parts [17].

### 3.1. Materials

**Figure 2:**
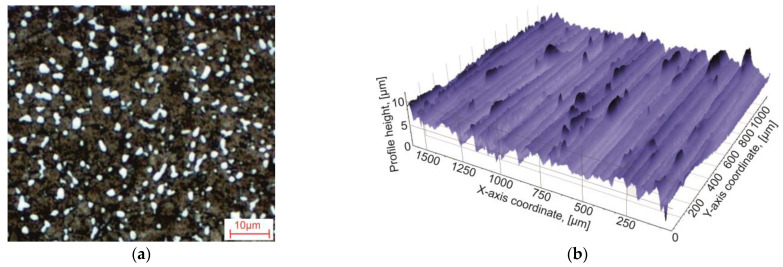
Initial characteristics of the machined titanium alloy Ti-1023 specimen: (**a**) initial metallographic microstructure and (**b**) initial surface topography of the machined specimen.

The machining of the test material by end milling was carried out at the machining center UWF 1202 H by Hermle—Figure 1. Titanium alloy Ti10V2Fe3Al (Ti-1023) was used as the tested material. The machined material was vacuum-annealed before cutting. The chemical composition of titanium alloy Ti-1023 is specified in Table 1, and its mechanical and thermal properties are given in Table 2. The initial characteristics of the machined titanium alloy Ti-1023 specimen are shown in Figure 2. The initial metallographic microstructure of the machined specimen is shown in Figure 2a. Figure 2b illustrates the initial surface topography of the machined specimen. The arithmetic mean profile height of the specimen initial surface was *R_a_* = 0.69 µm, and the maximum height of profile was *R_z_* = 3.74 µm.

The description of the experimental setup, the measurement equipment for the cutting force and torque components, the used tool (carbide end cutter) and its geometrical parameters, and the initial geometry of the machined workpiece (tested specimens) are described in a previously published study [84]. Table 2 shows the mechanical and thermal properties of the carbide end cutter. The milling was performed on a rectangular track along the cross-section of the workpiece in four levels, with a varying radial depth of cut *a_e_* of 0.5 mm, 1.0 mm, 1.5 mm, and 2.0 mm—Figure 1. The feed of the end milling cutter was changed from 0.06 mm/tooth to 0.12 mm/tooth in steps of 0.02 mm/tooth. The cutting speed *V_C_* was varied in five levels: 30 m/min, 45 m/min, 60 m/min, 90 m/min, and 120 m/min. The reliability of experimental values was ensured by repeating each set of cutting modes at least 5 times. The confidence interval was chosen to be equal to 0.9. The choice of confidence interval was based on analyzing the scatter of the individual experimental values of the cutting forces. Since there were no significant differences between the individual measured cutting forces, the average value was used as a representative value of the measured data. The maximum uncertainty in measuring the cutting forces was no more than 11%.

**Table 1:** Chemical composition of titanium alloy Ti10V2Fe3Al [85,86].

Material	Ti	Al	V	Fe	C	N	H	O	Other
Ti10V2Fe3Al	82.86–86.8%	2.6–3.4	9.0–11%	1.6–2.2%	<0.05%	<0.05%	<0.015%	<0.13%	≤0.3%

**Table 2:** Mechanical and thermal properties of titanium alloy Ti10V2Fe3Al and the milling cutter [85,87].

Material	Strength (MPa)		Elastic Modulus (GPa)	Elongation (%)	Hardness	Poisson′s Ratio	Specific Heat (J/kg·K)	Thermal Expansion (µm/m·°C)	Thermal Conductivity (W/m·K)
	Tensile	Yield							
Ti10V2Fe3Al	1282	1220	110	4–10	HV 430	0.35	527	9.7	7.0
Milling cutter	-	-	650	-	HV 1550	0.25	251	-	59

**Figure 3:**
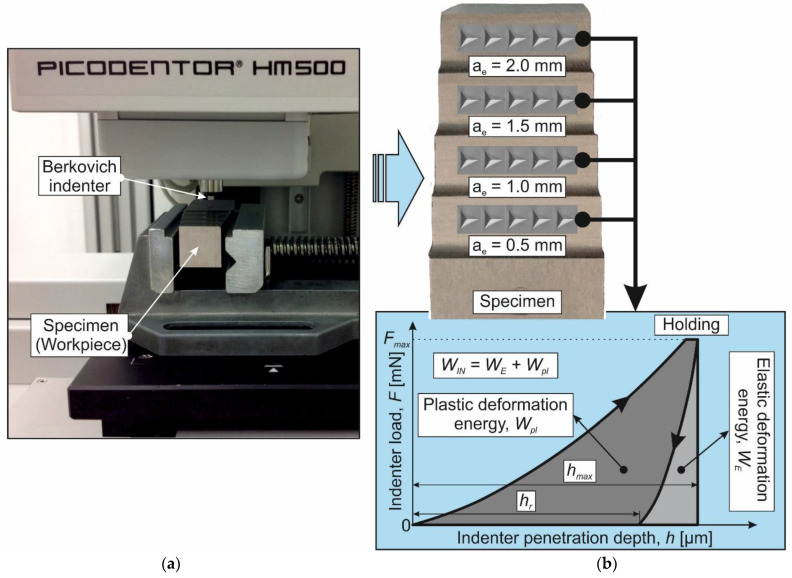
Experimental setup for measurements via instrumental nanoindentation and measurement scheme: (**a**) experimental setup for nanoindentation; (**b**) specimen image and instrumented indentation diagram.

The influence of the milling process on the mechanical characteristics of the machined workpieces’ subsurface layers was evaluated through the instrumented nanoindentation and sclerometry of the machined surfaces. The mechanical properties of the milled subsurface layers of the workpiece were evaluated through instrumented indentation using a Fischer Picodentor HM500 measuring system—Figure 3a. The instrumented indentation was performed on the milled surfaces of the specimen with different radial depths of cut *a_e_*—Figure 3b. The measurements were performed with a Berkovich indenter at a maximum force on the indenter equal to 450 mN. The load applied to the indenter was recorded using a force sensor. The accuracy of the indenter load was 0.02 mN, and the accuracy of the indenter depth measurement was 5 nm. At the same time, the depth of indenter penetration into the specimen was recorded by the position-measuring system. The change rate of the indenter load was 20 mN/s. Once the maximum indenter load was reached, the indenter was maintained at this load and then unloaded (see diagram in Figure 3b). The delay time was 5 s. The instrumented nanoindentation process was repeated at least 10 times for each set value of cutting speed *V_C_*, cutter feed *f,* and radial depth of cut *a_e_*. According to the instrumented nanoindentation diagram obtained as a test result, the Vickers HV microhardness of the subsurface layers, the indenter elastic penetration work *W_E_*, its plastic penetration work *W_pl,_* and total penetration work *W_IN_* were determined (see the diagram in Figure 3b). The determined values of the total indenter penetration energy were averaged over the tests performed. In this case, the measurement’s largest error was no more than 10%.

**Figure 4:**
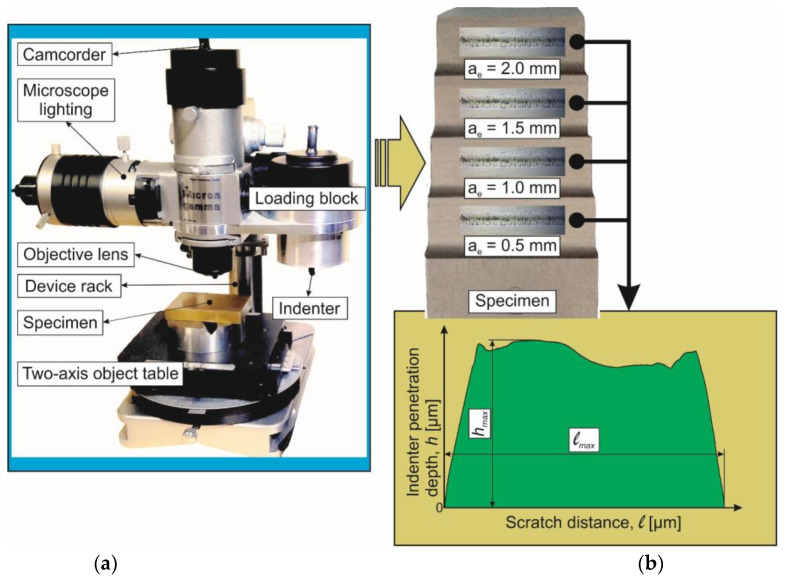
Experimental setup for sclerometry and its measurement scheme: (**a**) experimental setup for sclerometry of machined surfaces; (**b**) specimen image and sclerometry diagram.

The mechanical property evaluation of the milled subsurface layers was performed through sclerometry using the “Micron-gamma” device [17,55] at a Berkovich indenter load of 100 mN—Figure 4.

In this case, the indenter displacement velocity was 20 μm/s and the scratch length was about 630 µm. The Berkovich indenter was set so that the projection of one of its edges was parallel to the velocity vector of the indenter movement. Sclerometry tests were repeated at least 8 times for each set value of cutting speed *V_C_*, cutter feed *f,* and radial depth of cut *a_e_*. The error of the averaged values of the maximum indenter penetration depth along the scratch length (see the sclerometry diagram in Figure 4b) did not exceed 11%. To perform scratch analysis on the “Micron-gamma” device, an optical profilometer “Micron-alpha” was used [17,55]. The vertical resolution of the microtopography images of the machined surfaces obtained on this device was about 2 nm.

### 3.2. Methods

In the first part of the study on the mechanical property generation of the machined subsurface layers [17], the relationship between the conditions of the machining process and the mechanical characteristics of the above-mentioned subsurface layers is shown. Similar to the previously studied process of orthogonal cutting (see [17,55]), the milling process generates certain physical and mechanical characteristics in the subsurface layers of the workpiece due to the elastic–plastic interaction of the end cutter with the machined material and its subsequent separation into chips and the machined surface of the workpiece with the fracture of this material. Unlike orthogonal cutting, in which the stress–strain state of the machined material corresponds to a plane (two-dimensional), in the milling process, as in other real cutting processes, the machined material is in a spatial (three-dimensional) stress–strain state [88,89,90]. However, both in the spatial process stress–strain state (for the considered case in the milling process), and in the two-dimensional stress–strain state, the mechanical properties of the machined subsurface layers are determined to a significant degree by the cutting process conditions. In this case, either the adiabatic hardening of the machined material or its isothermal softening is realized in the cutting zones [91]. These conditions are also determined by the thermomechanical interaction (contact) between the end cutter and the machined material in the cutting zones. At the same time, the influence of contact conditions was evaluated by the well-proven energy characteristics of the cutting process for this purpose [17]. Taking into account the complexity and multiplicity of physical processes in the cutting zones during the spatial process of milling, the contact conditions of the end cutter with the machined material were estimated using the total cutting power *P_C_* and the plastic deformation work of the machined material in the tertiary cutting zone *A_cf_*. Thus, the mechanical characteristics of the machined subsurface layers generated during end milling, namely, microhardness and indenter penetration work, determined using instrumented nanoindentation, and maximum indenter penetration depth, determined using sclerometry, were considered according to the total cutting power *P_C_* and the plastic deformation work of the machined material in the tertiary cutting zone *A_cf_*. This is postulated by the following two statements:

- *The thermomechanical interaction of the end cutter with the machined workpiece is evaluated using the total milling power and is proportional to the indenter penetration work in the workpiece machined surface, determined using the instrumented nanoindentation of the machined subsurface layers, and proportional to the maximum depth of the indenter penetration in the subsurface layers, determined using the sclerometry of the machined subsurface layers:*
  (1) $$\underset{ℜ}{\forall }S_{C}\in ℜ\;\overset{S_{Cn}}{\underset{S_{C1}}{\exists }}\;P_{C}\;\propto \;W_{IN}\vee \;P_{C}\;\propto \;h_{\max}\;,$$
  where ℜ is the existence space of cutting process states (conditions); *S_C_* is the cutting process state; *P_C_* is the total cutting power; *W_IN_* is the total indenter penetration work through the instrumented nanoindentation of the milled surface; and *h_max_* is the maximum indenter penetration depth during the sclerometry of the milled surface.
- *The thermomechanical interaction of the end cutter with the machined workpiece is evaluated through the plastic deformation work of the machined material in the tertiary cutting zone during milling and is proportional to the indenter penetration work in the workpiece machined surface, determined using the instrumented nanoindentation of the machined subsurface layers, and proportional to the maximum depth of the indenter penetration in the subsurface layers, determined using the sclerometry of the machined subsurface layers:*
  (2) $$\underset{ℜ}{\forall }S_{C}\in ℜ\;\overset{S_{Cn}}{\underset{S_{C1}}{\exists }}\;A_{cf}\;\propto \;W_{IN}\vee \;A_{cf}\;\propto \;h_{\max}\;.$$

The total cutting power *P_C_* was determined according to the dependence known from cutting theory [88,89,92]:

(3) $$\;P_{C}\;=\;F_{C}\cdot V_{C},$$

where *F_C_* is the total cutting force, and *V_C_* is the cutting speed.

The cutting force *F_C_* was calculated as the resultant of the cutting force components measured during milling, as is presented in Figure 1 and Section 3.1. The plastic deformation work of the machined material in the tertiary cutting zone *A_cf_* was determined as a dependence function of the equivalent stresses *σ_cf_* acting in the machined material in the tertiary cutting zone on the strains *ε_cf_* of the machined material in this zone:

(4) $$\;A_{cf}\;=\;f(\sigma_{cf},\;\varepsilon_{cf})\;=\;V_{m}\cdot \int_{t_{s}}^{t_{e}}\sigma_{cf}d\varepsilon_{cf},$$

where *V_m_* is the material removal volume, and *t_s_* and *t_e_* are the simulation start and end times, respectively.

A determination of the dependence function (4) was performed by simulating the milling process of the titanium alloy Ti-1023 workpiece (see Section 3.2) with an end cutter using the previously developed numerical model of the milling process [84,86]. The cutting tool in these models was modeled as a perfectly rigid body and the workpiece material as an isotropic material defined by the Johnson–Cook constitutive equation [93,94]. The contact conditions between the tool and chip and between the tool and workpiece were specified via the Coulomb model [95]. Friction coefficients were determined according to a previously developed methodology [96]. In this case, the friction coefficient in the plastic area of the secondary cutting zone was *F_RFp_* = 0.786, the friction coefficient in the elastic area of the secondary cutting zone was *F_RFe_* = 0.405, and the friction coefficient in the tertiary cutting zone was *F_CF_* = 0.623. The fracture mechanism of the machined material [97] was realized using the Cockcroft and Latham model [98]. The critical stress value of the Cockcroft and Latham model as well as the parameters of the constitutive equation were found through sensitivity analysis by DOE (Design of Experiment) [84,86].

**Figure 5:**
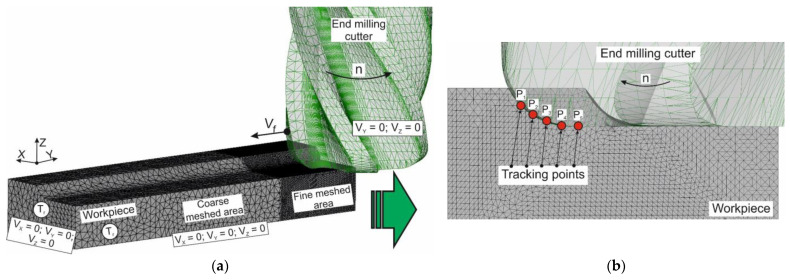
Layout scheme of tracking points: (**a**) initial geometric model of milling with a mesh and boundary conditions; (**b**) location of tracking points.

A determination of stresses and strains of the machined material in the region of the tertiary cutting zone through a simulation of the milling process with the end cutter was performed using tracking points. The layout of tracking points is shown in Figure 5. The five tracking points (P_1_, P_2_, P_3_, P_4_, and P_5_) located in the workpiece material in the tertiary cutting zone region were used. Before the plastic deformation work of the machined material in the tertiary cutting zone *A_cf_* was calculated, the stresses and strains determined at the indicated points were averaged over all five points.

## 4. Results and Discussion

**Figure 6:**
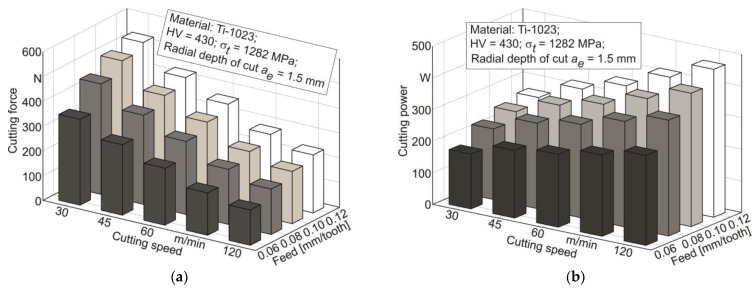
Dependence of the resultant cutting force and cutting power on cutting speed and cutter feed: (**a**) cutting mode influence on the resultant cutting force; (**b**) cutting mode influence on cutting power.

The influence of the milling process on the mechanical characteristics of the machined material subsurface layers was evaluated using the value of the resulting cutting force *F_C_* and cutting power *P_C_*, and the thermomechanical effect of the end cutter on the machined material *A_cf_* in the tertiary cutting zone (see Section 3.2). The dependence of the resultant milling force *F_C_* calculated from the measured values of the cutting force components and cutting power *P_C_* on the cutter feed and cutting speed is shown in Figure 6. The resulting cutting force increases proportionally as the cutter feed increases—Figure 6a.

The change in cutting force is explained with a proportional increase in the volume of removed material in chip form. At the same time, increasing the cutting speed leads to a significant reduction in the resultant cutting force—Figure 6a. This effect of cutting speed is explained by the predominant influence of machined material softening over its strain hardening [90,99,100]; in other words, the increase in cutting temperature caused by an increase in cutting speed softens the machined material to a greater extent than this material is hardened due to the speed factor. An increase in cutter feed also causes a corresponding increase in cutting power, as is presented in Figure 6b, due to the increased volume of workpiece material removed. In contrast to the effect on the resulting cutting force, an increase in cutting speed leads to a significant increase in cutting power. In all probability, this is caused by the numerical influence of cutting speed, since the value of cutting speed is included as a multiplier in the dependence of cutting power determination.

The determination of the thermomechanical work effect of the end cutter on the machined material *A_cf_* in the tertiary cutting zone was performed, as announced in Section 3, by simulating the stresses in the machined material and their corresponding strains. The adequacy of the developed numerical model of titanium alloy Ti-1023 milling for the studied range of cutting modes was checked by comparing the experimentally determined values of the resulting cutting force *F_C_* and cutting power *P_C_* with the corresponding simulated values, as was presented in Section 3.

**Figure 7:**
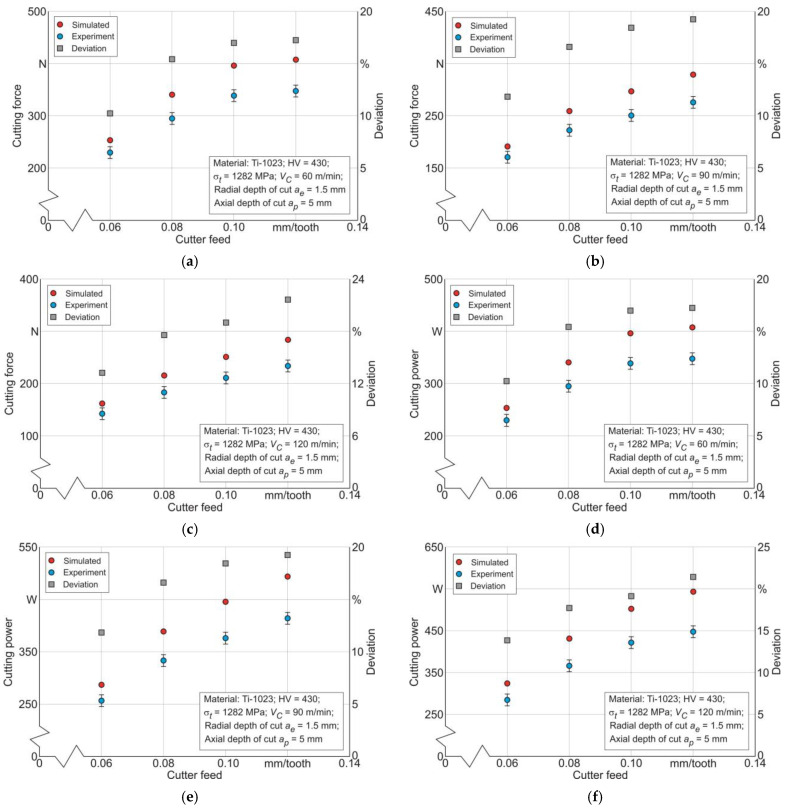
Comparison of experimental and simulated values of the resultant cutting force and cutting power with changing cutting modes: (**a**) cutting force dependence on cutter feed at cutting speed *V_C_* = 60 m/min; (**b**) cutting force dependence on cutter feed at cutting speed *V_C_* = 90 m/min; (**c**) cutting force dependence on cutter feed at cutting speed *V_C_* = 120 m/min; (**d**) cutting power dependence on cutter feed at cutting speed *V_C_* = 60 m/min; (**e**) cutting power dependence on cutter feed at cutting speed *V_C_* = 90 m/min; and (**f**) cutting power dependence on cutter feed at cutting speed *V_C_* = 120 m/min.

The results of this comparison, exemplarily for cutting speeds from 60 m/min to 120 m/min, and for a radial depth of cut *a_e_* = 1.5 mm and an axial depth of cut *a_p_* = 5 mm, are shown in Figure 7. Together with the cutting force and cutting power values, the deviation values between the experimental and simulated values are presented in the figure. The specified deviations for a cutting speed *V_C_* = 60 m/min and the entire range of cutter feed variation lie between about 10.3% and about 17.3%, as is presented in Figure 7a,d. The corresponding deviations between the experimental values of cutting force and cutting power for a cutting speed *V_C_* = 90 m/min and the entire range of cutter feed range lie between about 11.9% and about 19.3%, as is presented in Figure 7b,e, and for a cutting speed *V_C_* = 120 m/min and the entire range of cutter feed range, they lie between about 13.9% and about 21.5%, as is presented in Figure 7c,f. Thus, it can be assumed that the numerical model of titanium alloy milling is able to adequately simulate the characteristics of the machining process used later to match them with the mechanical characteristics of the machined subsurface layers of the workpiece.

**Figure 8:**
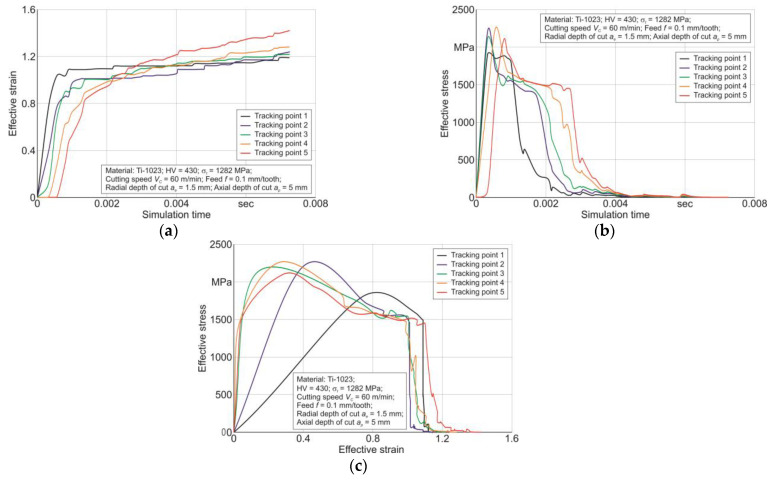
Change in effective stress and effective strain in the region of the tertiary cutting zone: (**a**) effective strain variation over the simulation time; (**b**) effective stress variation over the simulation time; (**c**) relationship between effective stress and effective strain for five tracking points.

The stresses and strains of the machined material in the tertiary cutting zone region, which are further used to calculate the *A_cf_* thermomechanical impact of the end cutter on the machined material, were determined using five tracking points (see the diagram in Figure 5 and Section 3.2) as a result of the milling process simulation. The variations in effective strain *ε_cf_* and effective stress *σ_cf_*, determined at five specified tracking points, by simulation time and the dependence of effective stress *σ_cf_* on effective strain *ε_cf_*, exemplarily for a cutting speed *V_C_* = 60 m/min and a radial depth of cut *a_e_* = 1.5 mm, are presented in Figure 8.

**Figure 9:**
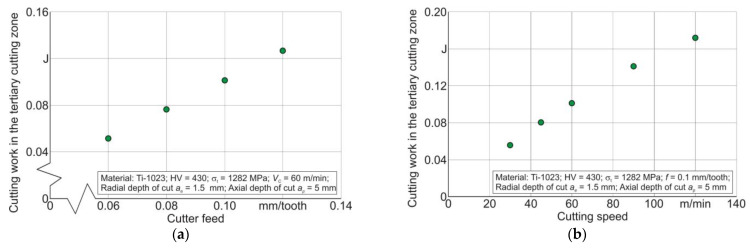
Effect of cutting modes on cutting work *A_cf_* in the region of the tertiary cutting zone: (**a**) effect of cutter feed on cutting work; (**b**) effect of cutting speed on cutting work.

The variation in effective strain *ε_cf_* and effective stress *σ_cf_* by simulation time for the considered five tracking points placed in the tertiary cutting zone region is presented in Figure 8a,b. According to the results of the change in *ε_cf_* and *σ_cf_* their relationship is determined and presented in Figure 8c. This relationship together with the material volume value *V_m_* removed as a result of the milling, determined for the studied range of cutting modes, was used to calculate the cutting work *A_cf_* in the tertiary cutting zone (see Equation (4), Section 3). In this case, the volume *V_m_* was determined from the nominal chip shape, without taking chip compression into account. This assumption was made on the basis that the chip compression ratio of the used Ti-1023 titanium alloy for the studied range of cutting modes does not exceed 1.1 [84,86]. The effect of cutter feed and cutting speed on the cutting work *A_cf_* is presented exemplarily for a cutting speed *V_C_* = 60 m/min and a radial depth of cut *a_e_* = 1.5 mm in Figure 9. The cutting work *A_cf_* in the tertiary cutting zone increases almost linearly as the cutter feed increases from 0.06 mm/tooth to 0.12 mm/tooth—Figure 9a. This increase in cutting work seems logical because as the cutter feed rate increases, the amount of material removed per unit of time increases. An increase in cutting speed in the studied range causes a monotonic increase in cutting work *A_cf_*—Figure 9b. This increase in *A_cf_* is most likely also a consequence of the increase in the material removal volume with increasing cutting speed.

**Figure 10:**
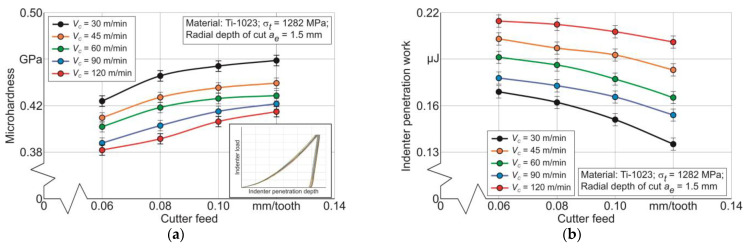
Effect of cutter feed and cutting speed on microhardness of machined subsurface layers of the workpiece and total indenter penetration work: (**a**) microhardness dependence on cutting modes; (**b**) indenter penetration work dependence on cutting modes.

The values that predetermine the mechanical characteristics of the workpiece subsurface layers machined using end milling are the microhardness of these layers, the total indentation work, and the maximum indentation depth, as is presented in Figure 1 and Section 3. The first two values are determined by instrumented nanoindentation for a wide range of cutting conditions, as is presented in Figure 3 and Section 3.1. The results of the instrumented nanoindentation are exemplified by the radial depth of cut *a_e_* = 1.5 mm in Figure 10. This figure demonstrates the effect of cutter feed and cutting speed on the microhardness of the milled subsurface layers and the total indenter penetration work. The microhardness of the machined subsurface layers monotonically increases with increasing cutter feed—Figure 10a. This effect of tool feed is logical because as the feed increases, the material removed volume per unit of time increases. The increase in the volume of removed material entails a corresponding increase in the strain degree of the machined subsurface layers of the workpiece, which leads in turn to the hardening of these layers and, naturally, to an increase in their microhardness [88,99,100]. At the same time, the microhardness of the subsurface layers decreases with increasing cutting speed—Figure 10a. In all probability, the reason for such an effect of cutting speed on microhardness is the increase in cutting temperature with increasing cutting speed, which entails the softening of subsurface layers [89,90,100]. The total indenter penetration work *W_IN_* monotonically decreases with increasing cutter feed—Figure 10b. Such an effect of feed is quite understandable, since the consequence of increasing the cutter feed is (as already shown above) a corresponding increase in the volume of material removed per unit of time, entailing an increase in the strain degree of the machined material layers, and hence an increase in the hardening of these layers [89,90,92]. In turn, an increase in the hardening of the subsurface layers leads to a lower degree of indenter penetration into the studied material and, as a consequence, to a lower indenter penetration work *W_IN_*. In this case, the consequence of increasing cutting speed is a monotonic increase in the indenter penetration work—Figure 10b. This increase is due to the softening of the machined subsurface layers of the workpiece caused by the increase in cutting temperature with increasing cutting speed [99,101]. The similar character of changes in the studied mechanical characteristics of subsurface layers is also observed at other values of the radial depth of cut *a_e_*.

**Figure 11:**
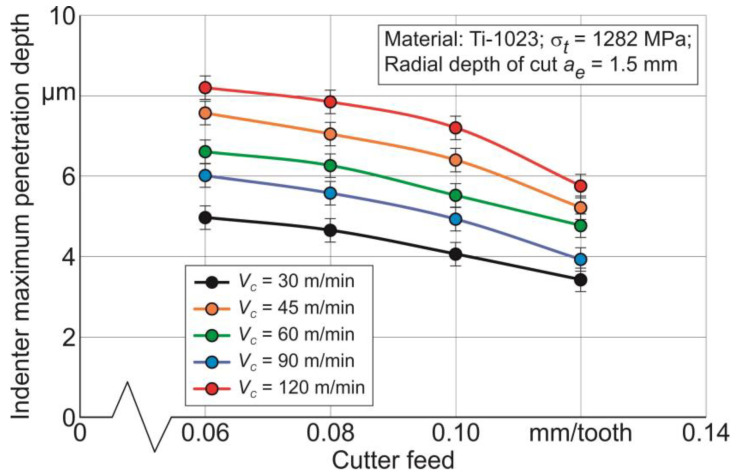
Indenter penetration depth dependence on cutter feed and cutting speed.

The maximum indenter penetration depth was determined by the sclerometry of the specimen’s milled surface, as is presented in Figure 4 and Section 3.1. The results of the sclerometry analysis are shown exemplarily for the radial depth of cut *a_e_* = 1.5 mm in Figure 11.

The maximum depth of indenter penetration *h_max_* into the milled surface as a result of sclerometry monotonically decreases with the cutter feed increase. This is due to the increased hardening of the workpiece subsurface layers as a result of machining with increasing cutter feed due to the corresponding increase in the volume of material removed per unit of time [99,100]. As a result of this increase in the hardening of the subsurface layers, the indenter penetration depth at constant load decreases [17]. At the same time, the maximum depth of indenter penetration increases with increasing cutting speed—Figure 11. This effect of cutting speed is a consequence of softening at the workpiece milled subsurface layers [88,89,99], caused by the increase in cutting temperature as a result of increasing cutting speed [89,101].

The evaluation of the mechanical characteristics of the milled subsurface layers, performed through instrumented nanoindentation and sclerometry with a significant change in cutting modes (cutter feed, cutting speed, and radial depth of cut), indicates the predominant effect of the milling process characteristics on the studied mechanical characteristics. This indicates the existence of a close correlation between the characteristics of the milling process and the mechanical characteristics of the machined subsurface layers. The presence of such a relationship makes it possible to compile the characteristics of the milling process with the studied mechanical characteristics according to the above-formulated postulates, as is presented in Section 3, Equations (1) and (2). To generalize these comparisons, the milling process characteristics should serve their energy values. In this case, such characteristics are cutting power and the work of thermomechanical interaction between the cutter and the workpiece in the tertiary cutting zone.

**Figure 12:**
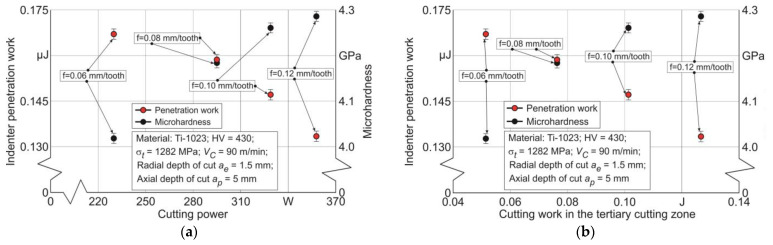
The coincidence of the indenter penetration work and microhardness with the cutting power and cutting work in the tertiary cutting zone: (**a**) depending on the cutting power; (**b**) depending on the cutting work in the tertiary cutting zone.

The coincidence results of both mechanical characteristics of the machined subsurface layers, indenter penetration work and microhardness, with cutting power *P_C_* and cutting work *A_cf_* in the tertiary zone are presented exemplarily for a cutting speed *V_C_* = 60 m/min and a radial depth of cut *a_e_* = 1.5 in Figure 12.

With increasing cutting power, the indenter penetration work *W_IN_* monotonically decreases, and similarly, the microhardness of the machined subsurface layers of the workpiece increases with increasing cutter feed—Figure 12a. A similar effect on indenter penetration work and microhardness is due to the cutting work in the tertiary cutting zone region *A_cf_*—Figure 12b. This influence effect can be explained by the fact that the increase in the interaction energy between the cutter and the machined material as a result of the increase in cutting power and cutting work in the tertiary cutting zone contributes to the hardening of the machined subsurface layers. The increased hardening of these layers inhibits indenter penetration, resulting in the observed increase in microhardness and the decrease in indenter penetration work.

**Figure 13:**
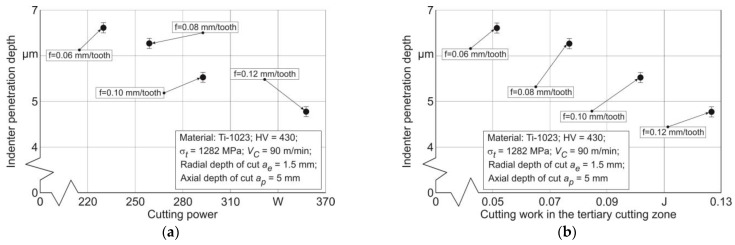
The coincidence of indenter penetration depth during sclerometry with the cutting power and cutting work in the tertiary cutting zone: (**a**) depending on the cutting power; (**b**) depending on the cutting work in the tertiary cutting zone.

When comparing the maximum indenter penetration depth *h_max_* with the energy characteristics of the cutting process, with cutting power *P_C_* and cutting work *A_cf_* in the tertiary cutting zone, a monotonic decrease in *h_max_* value is observed—Figure 13. This decrease occurs in conjunction with an increase in the indicated energy characteristics caused by an increase in cutter feed. The decrease in *h_max_* with increasing cutting power *P_C_* and cutting work *A_cf_* in the tertiary cutting zone is also explained by the hardening of the milled subsurface layers of the workpiece. The increase in the hardening degree of these layers causes a decrease in the possibility of indenter penetration into the workpiece milled surface.

The analysis of the coincidence of the mechanical characteristics from the milled subsurface layers with the characteristics of the cutting process makes it possible to confirm the above-formulated postulates about the significant influence of the considered energy characteristics of the end milling process on the studied integral mechanical characteristics. This creates the possibility of a purposeful selection of the contact conditions between the tool and the machined material through the assignment of cutting modes that ensure the necessary mechanical characteristics of the workpiece subsurface layers. The achievement of the specified mechanical characteristics of the machined by the milling subsurface layers may in turn enable the required service properties of machine parts.

## 5. Conclusions

The research performed here is devoted to the formation patterns of subsurface layers mechanical characteristics of titanium alloy Ti10V2Fe3Al (Ti-1023) workpieces as a result of their milling by an end milling cutter. The mentioned patterns are considered based on an analysis of the coincidence of the measured mechanical characteristics with the milling process characteristics. To generalize the studied patterns, integral characteristics were used as mechanical characteristics: indenter penetration work in the machined surface, the microhardness of these surfaces, and the maximum depth of indenter penetration. The measurements of the mentioned integral characteristics were carried out using nondestructive testing methods, namely, the instrumented nanoindentation and sclerometry of the studied subsurface layers. As generalized characteristics of the milling process, cutting power and the thermomechanical interaction work of the cutter with the machined material in the region of the tertiary cutting zone were used. To determine the cutting power of the milling process, the measured value of the resultant cutting force was used, and the thermomechanical interaction work of the cutter with the machined material was calculated using a numerical model of the milling process.

The following patterns of changes in the milling process kinetic characteristics of titanium alloy Ti-1023 and mechanical characteristics of the machined subsurface layers depending on the machining modes were established by experimental studies:

➢ The resulting cutting force monotonically increases with increasing cutter feed and decreases monotonically with increasing cutting speed;
➢ The microhardness of the workpiece subsurface layers monotonically increases with increasing cutter feed and decreases with increasing cutting speed;
➢ The indenter penetration work as a result of the instrumented nanoindentation of the workpiece subsurface layers decreases with increasing cutter feed and increases with increasing cutting speed;
➢ The indenter maximum penetration depth as a result of the sclerometry of the workpiece subsurface layers decreases with increasing cutter feed and increases with increasing cutting speed.

The machining modes’ influence on the generalized characteristics of the cutting process is characterized by the following patterns:

➢ The cutting power increases both with increasing cutting speed and with increasing cutter feed;
➢ The cutting work in the tertiary cutting zone increases both with increasing cutter feed and with increasing cutting speed.

The regularities established as a result of experimental and simulation studies of the end tool milling process enable the possibility of identifying the coincidence of the machining process energy characteristics with the integral mechanical characteristics of the milled subsurface layers. The use of cutting process energy characteristics for this purpose provides a numerical characterization of machining technology as a way of generating mechanical characteristics of subsurface layers. This, in turn, ensures the possibility of analyzing the impact of the machining technology regardless of the technology used.

The analysis of the coincidence of the integral mechanical characteristics of the workpiece milled subsurface layers with the cutting process energy characteristics provides the possibility of a purposeful selection of the milling process conditions with the end tool of titanium alloy Ti-1023, conditioned by the appropriate choice of cutting modes. In turn, the possibility of such a choice enables the achievement of the required service properties of manufactured parts with mechanical characteristics of subsurface layers generated by the milling process.

The research direction presented in the paper is planned to be further developed by studying the coincidence of the characteristics of other machining processes with the mechanical characteristics of the machined subsurface layers. In addition, it is planned to expand the type of machined materials.

## Data Availability

Data are contained within the article.
